# The impact of low-dose glucocorticoids on disease activity, bone mineral density, fragility fractures, and 10-year probability of fractures in patients with rheumatoid arthritis

**DOI:** 10.1136/jim-2018-000723

**Published:** 2018-06-10

**Authors:** Tien-Tsai Cheng, Han-Ming Lai, Shan-Fu Yu, Wen-Chan Chiu, Chung-Yuan Hsu, Jia-Feng Chen, Ying-Chou Chen

**Affiliations:** 1 Kaohsiung Chang Gung Memorial Hospital, Kaohsiung, Taiwan; 2 Chang Gung University College of Medicine, Kaohsiung, Taiwan

**Keywords:** osteoporosis, osteoporotic fractures

## Abstract

This study aimed to investigate the effect of low-dose glucocorticoids (LDGs) on disease activity, bone density, and fractures in patients with rheumatoid arthritis (RA). This was an interim analysis of the RA Registry. Demographic data and clinical characteristics, including fracture risk assessment tool, were collected. 25(OH) Vitamin D, bone mineral density (BMD), and intact parathyroid hormone were measured at enrollment. The study group were those who took LDGs (2.5–7.5 mg/day prednisolone or equivalent dose), and the others were included as the control group. A total of 425 participants were enrolled, including 85 (20%) in the control group and 340 (80%) in the study group. The demographics and clinical characteristics were comparable between the two groups. Compared with the control group, the LDGs group had a significantly lower vertebral BMD (L 1–4) (g/cm^2^), (0.854 vs 0.896, p=0.046), significantly higher rate of previous fractures (103 (30.3%) vs 13 (15.3%), p=0.006), higher 10-year probability of major fractures (14 (15.5) vs 8 (8.6), p<0.0001), and higher 10-year probability of hip fractures (4.4 (8.4) vs 2 (3.9), p<0.0001). Disease activity appeared to be similar in the patients with RA regardless of whether or not they received LDG treatment. However, the patients with RA who received LDG treatment had a lower BMD at the spine (L1–4) and a higher rate of previous fractures that was associated with a significantly higher 10-year probability of fractures than those who did not receive LDG treatment.

**Signiﬁcance of this study**

**What is already known about this subject?**

- Rheumatoid arthritis (RA) is often treated with glucocorticoids (GCs).
- GC therapy had impact on risk of fractures and low bone mineral density (BMD), especially of the trabecular bone.

**What are the new ﬁndings?**

- Lower BMD at the spine (L1–4) and a higher rate of previous fractures in patients with chronic RA who received low-dose glucocorticoid (LDG) treatment that was associated with a significantly higher 10-year probability of fractures than those who did not receive LDG treatment.

**How might these results change the focus of research or clinical practice?**

- It is mandatory to carefully assess and detect fractures in patients with RA who receive chronic GC therapy.
- We recommend that physicians should assess and determine the risk of fractures in patients with RA who receive chronic GC therapy.

## Introduction

Rheumatoid arthritis (RA) is often treated with glucocorticoids (GCs).^1^ GC therapy is considered to have association with the risk of fractures^2^ and the rapid reduction in the bone mineral density (BMD), especially of the trabecular bone. It is reported that after 5–7 months of GC therapy, 25% of them had loss of trabecular bone.^3^ Besides, among the patients with secondary osteoporosis, GC treatment is the most common,^1^ which leads to increase in the risk of fractures. Van Staa *et al* 4 reported a twofold increased risk of fractures in postmenopausal women taking GCs.

Previous studies have identified the factors that contribute to the risk of fractures. The WHO then approved the development of aflow chart for risk assessment that can be used in primary care settings, and when BMD is not available. So, the fracture risk assessment tool (FRAX) was developed, which can be used to assess 10-year probability of major osteoporotic fracture (clinical spine, forearm, hip, or shoulder) and a hip fracture in both men and women.^5 6^ GC therapy for RA leads to fractures and impacts mortality,^7 8^ the patient’s quality-of-life (including physical functioning),^9 10^ and the risk of future fractures.^11^

A few studies have investigated the effect of chronic GC therapy on osteoporosis in patients with RA.

The primary objective of this study was to determine the BMD in these patients, and the secondary objective was to determine the risk of fractures (major and minor).

## Materials and Methods

This was an interim analysis of the RA Registry conducted at Chang Gung Memorial Hospital in Kaohsiung (CGMHK). All participants provided written informed consent.

In the Registry, consecutive RA study subjects who visited Rheumatology Clinic at CGMHK from September 1, 2014 to September 2017 and fulfilled classification criteria for RA were enrolled.^12^

The demographic data and clinical characteristics, including lifestyle, including diet, evidence of previous fragility fracture, and risk factors for fracture in FRAX, were collected. Anti-citrullinated protein antibodies (anti-CCP), rheumatoid factor (RF), erythrocyte sedimentation rate (ESR), disease activity-28 joint-erythrocyte sedimentation rate score (DAS28-ESR), C reactive protein (CRP), 25(OH) Vitamin D, intact parathyroid hormone, and BMD weres measured at enrollment.

Those on low-dose glucocorticoid (LDG, 2.5–7.5 mg/day prednisolone or equivalent dose) were included as the study group and the others were included as the control group. Those with GC intake of more than 7.5 mg/day were excluded. We calculated the 10-year probabilities of major and hip fractures using the Taiwanese-specific FRAX. Participants were categorized into low-risk, medium-risk, and high-risk groups for major osteoporotic fracture based on the 10-year fracture risk probability calculated using FRAX with cut-off points of 10% and 20%.^13^ We followed the guidance of the International Osteoporosis Fundation (IOF)/European Calcified Tissue Society (ECTS) for glucocorticoid induced osteoporosis (GIOP) to calculate the intervention threshold for each participant.^14^ We modified the calculation of individual intervention threshold (IIT) by inputting gender, body weight, and body height and assumed that the participant had a ‘prior fracture’ but no other risk factors and BMD unavailable.^15^ Then, we recalculated the individual-specific 10-year probability of major fracture of that participant by inputting the real situation. We defined ‘above IIT’ as 10-year probability of major fracture of that participant being higher or equal to IIT of the same participant. We compared the proportion of the above IIT between the GC users and controls.^15^

## Results

There were a total of 425 study subjects. There were 85 (20%) who never used corticosteroids (control group) and 340 (80%) who accepted LDG (study group). The age, BMD, disease duration, symptoms of RA to diagnosis of RA, habit (tea and coffee), proportion of vegetarian, anti-CCP, RF, ESR, CRP, DAS28, and vitamin D level were the same for both groups. The demographics and clinical characteristics were comparable between the groups. Compared with control group subjects, study subjects on LDG had a significantly lower vertebral BMD (L 1–4) (g/cm^2^), (0.854 (0.200) vs 0.896 (0.201), p=0.046) and a higher rate of previous fracture (103 (30.3) vs 13 (15.3), p=0.006) (table 1). The 10-year probabilities (with BMD (without BMD)) of major (14 (15.5) vs 8 (8.6), p<0.0001) and hip (4.4 (8.4) vs 2 (3.9), p<0.0001) fractures, respectively, were higher in LDG group (table 2).

**Table 1:** Participants’ demographics and clinical characteristics

Variables*	All n=425	LDG (–) n=85	LDG (+) n=340	P values
Age (years)	57 (14)	58 (14)	57 (14)	0.9107
Female, n (%)	375 (88.2)	75 (88.2)	300 (88.2)	1
BMI (kg/m^2^)	23.4 (5.0)	23.4 (4.7)	23.4 (5.0)	0.916
Disease duration (years)	10 (13) n=408	12 (13) n=83	10 (13) n=325	0.478
Symptom to diagnosis (years)	2 (5) n=408	1 (4) n=83	2 (5) n=325	0.336
Tea†, n (%)	81 (19.1)	17 (20.0)	64 (18.8)	0.805
Coffee†, n (%)	79 (18.6)	19 (22.4)	60 (17.6)	0.319
Vegetarian‡, n (%)	23 (5.4)	5 (5.9)	18 (5.3)	0.791
Anti-CCP (≥7 U/mL), n (%)	287 (69.3) n=414	51 (61.4) n=83	236 (71.3) n=331	0.082
RF (≥15 U/mL), n (%)	284 (67.5) n=421	57 (67.1) n=85	227 (67.6) n=336	0.930
ESR (mm/hour)	16 (20)	14 (18)	17 (21)	0.426
CRP (mg/L)	2.8 (6.7)	1.9 (4.2)	3 (7.4)	0.299
DAS28-ESR	3.2 (1.6) n=422	2.9 (1.7) n=84	3.3 (1.6) n=338	0.107
iPTH (pg/mL)	57 (14)	41.3 (23.3)	39.9 (28.2)	0.454
Vitamin D 25(OH) (ng/mL)	20.9 (9.3) n=332	22.0 (8.6) n=71	20.8 (10.0) n=261	0.679
Comorbidity, n (%)	238 (56)	41 (48.2)	197 (57.9)	0.107
bDMARD, n (%)	74 (17.4)	20 (23.5)	54 (15.9)	0.096
Osteoporosis¶, n (%)	109 (27.3) n=400	18 (22.2) n=81	91 (28.5) n=319	0.2551
Current treatment**, n (%)	62 (14.6)	6 (7.1)	56 (16.5)	0.028
Fall in previous year, n (%)	67 (16.4) n=409	11 (13.3) n=83	56 (17.2) n=326	0.388
Fracture, n (%)	116 (27.3)	13 (15.3)	103 (30.3)	0.006

*All data are expressed as median (IQR).

†, regular daily drinker; ‡, more than 3 years; ¶, femoral neck T-score ≤ −2.5; **, receiving antiosteoporosis therapy; n, data available.

Anti-CCP, anti-citrullinated protein antibodies; bDMARDs, biologic disease-modifying antirheumatic drugs; BMD, bone mineral density; BMI, body mass index; CRP, C reactive protein; DAS28-ESR, disease activity score-28 joint-erythrocyte sedimentation rate; ESR, erythrocyte sedimentation rate; iPTH, intact parathyroid hormone; LDG, low-dose glucocorticoid; RF, rheumatoid factor.

**Table 2:** Comparison of the difference of BMD and FRAX of low-dose steroid group or those without low-dose steroid group

	All n=425	LDG (–) n=85	LDG (+) n=340	P values
BMD (g/cm^2^)				
FN^c^	0.629 (0.137) n=400	0.636 (0.131) n=81	0.627 (0.141) n=319	0.314
Hip (total)	0.785 (0.180) n=400	0.779 (0.176) n=81	0.786 (0.181) n=319	0.731
L1–4	0.865 (0.197) n=403	0.896 (0.201) n=82	0.854 (0.200) n=321	0.046
Fracture, n (%)	116 (27.3)	13 (15.3)	103 (30.3)	0.006
FRAX (major, with BMD)	12.5 (14.3) n=400	8 (8.6) n=81	14 (15.5) n=319	<0.0001
FRAX (hip, with BMD)	3.5 (7.2) n=400	2 (3.9) n=81	4.4 (8.4) n=319	<0.0001
FRAX (major, no BMD)	11 (14.3)	7.1 (7.4)	12 (15.7)	<0.0001
FRAX (hip, no BMD)	3.2 (6.8)	1.6 (3.5)	3.6 (8.3)	<0.0001
FRAX (major, IT)	9.7 (9.1)	9.6 (8.3)	9.7 (9.0)	0.4556
FRAX (hip, IT)	2.5 (4.4)	2.3 (4.1)	2.6 (4.4)	0.4255

BMD, bone mineral density; FN, femoral neck; FRAX, fracture risk assessment tool; IT, intervention threshold; LDG, low -dose glucocorticoid.

## Discussion

Higher 10-year risk of major osteoporotic fracture or hip fracture was found in patients who received LDG compared with patients who did not receive steroid treatment, as determined by FRAX analysis. In addition, the 10-year risk of fractures in low-dose steroid treatment patients appeared to be particularly high. LDG has been associated with an increased risk of fractures,^16 17^ and GC administration has been reported to be a cause of secondary osteoporosis.^1^ Angeli *et al* 18 found that RA may increase the risk of fractures, and Van Staa *et al* 19 revealed that a combination of RA activity and GC therapy contributes to an increased risk of fractures. However, it is difficult to evaluate the risk of fractures with LDG therapy. In our study, chronic LDG treatment was associated with a higher risk of fractures. FRAX is mandatory because this method only includes data on whether or not the patient uses steroids and does not consider the impact of the cumulative GC dose on the risk of fractures. Hence, the fracture risk prediction according to FRAX may be augmented by including the chronicity and dosage of GC therapy. Therefore, the links between chronicity and dosage of GC therapy can lead to increased risk of fractures.^20^

The reason why GC therapy increases the risk of fractures may be because GC therapy reduces BMD^21 22^ and also decreases bone quality through microarchitectural changes.^22^ Thus, prophylactic treatment may improve bone quality as well as quantity.

The FRAX estimates the risk of fractures by clinical risk factors. The methods can be used alone or with BMD to improve the prediction of fracture risk. Several previous reports have developed methods to predict fracture risk by combining clinical risk factors and BMD,^23–25^ with risk factors including activities of daily living, liability to falls, impaired cognition, poor overall health, history of stroke, seizure disorder, and several different medications.

The particular feature of the FRAX is that it takes into account risk factors from RA and previous osteoporotic fractures.^26 27^ So, smoking, low BMD, and RA are risk factors for fractures. Therefore, at low T-scores, the risk of hip fractures lowers with age, in part due to the higher fractures associated with lower BMD values.

There are several limitations to this study. First, self-reported data contribute to all of the fractures. Second, a higher proportion of the patients were receiving medications to reduce the risk of fractures, and it may be regarded as a limitation when determining the prevalence of fractures in patients with RA. Third, a non-response bias may have impact on the results. It may lead to underestimation of absolute fracture risk. The analyses also have significant limitations that relate to risk factors and the outcome variables. The reason for which was an osteoporotic fracture which was not the same in all subjects, and the effects of this inconsistency are possibly to weaken the relationships that were found. Further problems lead to the questions to assess the presence or absence of risk factors, which varied between cohorts. These included questions on family history, prior fracture, smoking, and GC use. Recall bias and the validity of self-reported alcohol intake that are notoriously unreliable are particularly problematic in the elderly. Moreover, this was a single-center study. These limitations restrict the generalizability of the results. Nevertheless, this study has several strengths, including a large sample size relative to comparable studies. This approach is likely to have improved the accuracy to estimate the risk of fractures, to allow for more precise determination of the impact on patients’ physical functioning.

## Conclusions

We reported a relatively higher proportion of fractures in patients with RA receiving LDG. Our data also revealed that chronic GC therapy leads to non-vertebral fractures. Nevertheless, the risk of sustaining a fracture by the impact of cumulative GC is inconclusive. Besides, fractures had a negative effect on further fractures in a 10-year period. To care for patients with RA who receive chronic GC therapy, it is particularly important to carefully monitor to detect fractures. Also, we recommend to take preventative measures in patients with RA on chronic GC therapy to decrease the risk of fractures.
